# Body composition predictors of mortality in patients undergoing surgery for long bone metastases

**DOI:** 10.1002/jso.26793

**Published:** 2022-01-13

**Authors:** Olivier Q. Groot, Michiel E.R. Bongers, Colleen G. Buckless, Peter K. Twining, Neal D. Kapoor, Stein J. Janssen, Joseph H. Schwab, Martin Torriani, Miriam A. Bredella

**Affiliations:** ^1^ Department of Orthopaedic Surgery—Orthopaedic Oncology Service Massachusetts General Hospital—Harvard Medical School Boston Massachusetts USA; ^2^ Division of Musculoskeletal Imaging and Intervention, Department of Radiology Massachusetts General Hospital and Harvard Medical School Boston Massachusetts USA; ^3^ Department of Orthopedic Surgery, Amsterdam Movement Sciences Amsterdam University Medical Center—University of Amsterdam Meibergdreef Amsterdam The Netherlands

**Keywords:** body composition, computed tomography (CT), long bone metastases, mortality, sarcopenia

## Abstract

**Background and Objectives:**

Body composition measurements using computed tomography (CT) may serve as imaging biomarkers of survival in patients with and without cancer. This study assesses whether body composition measurements obtained on abdominal CTs are independently associated with 90‐day and 1‐year mortality in patients with long‐bone metastases undergoing surgery.

**Methods:**

This single institutional retrospective study included 212 patients who had undergone surgery for long‐bone metastases and had a CT of the abdomen within 90 days before surgery. Quantification of cross‐sectional areas (CSA) and CT attenuation of abdominal subcutaneous adipose tissue, visceral adipose tissue, and paraspinous and abdominal muscles were performed at L4. Multivariate Cox proportional‐hazards analyses were performed.

**Results:**

Sarcopenia was independently associated with 90‐day mortality (hazard ratio [HR] = 1.87; 95% confidence interval [CI] = 1.11–3.16; *p* = 0.019) and 1‐year mortality (HR = 1.50; 95% CI = 1.02–2.19; *p* = 0.038) in multivariate analysis while controlling for clinical variables such as primary tumors, comorbidities, and chemotherapy. Abdominal fat CSAs and muscle attenuation were not associated with mortality.

**Conclusions:**

The presence of sarcopenia assessed by CT is predictive of 90‐day and 1‐year mortality in patients undergoing surgery for long‐bone metastases. This body composition measurement can be used as novel imaging biomarker supplementing existing prognostic tools to optimize patient selection for surgery and improve shared decision making.

## 1 INTRODUCTION

Long bones are a common site for metastatic disease, especially in patients with advanced neoplastic disease.^1^, ^2^ Metastases to long bone compromise the structural integrity of the bone and its ability for load‐bearing, which can initially lead to painful microfractures followed by pathological fractures, which are associated with a decline in quality of life.^2^ Surgical stabilization is often performed for patients with pathological fractures of long bones, but prophylactic stabilization is also regularly considered for patients with known metastatic disease at high risk for a fracture. Due to the incurable nature of metastatic disease, treatment for these patients is primarily performed for palliative measures to maintain or optimize quality of life. For some patients, the benefits of surgery may not outweigh the disadvantages that come with it such as perioperative mortality, postoperative complications, hospitalization, and reoperations.^3^, ^4^ Less intensive treatment, such as radiation therapy or minimally invasive stabilization, might be more appropriate for patients with an estimated short survival. Expected survival is thus an important factor in decision making of the most‐appropriate therapy.^5^, ^6^ Many studies assess clinical factors which are associated with survival in patients with long bone metastases and some studies incorporate these factors in prediction tools.^7^, ^8^, ^9^, ^10^, ^11^ However, we are not aware of studies that consider computed tomography (CT) measurements of body compositions as predictors.

Patients with long bone metastases routinely undergo CTs for staging, assessment of treatment response, or surveillance. These CTs are readily available for analysis and body composition measures could potentially serve as imaging biomarkers to predict outcome in this population without additional risk. Recent studies have proposed CT body composition measurements of muscle and fat depots as biomarkers for survival in patients with and without malignant disease.^12^, ^13^, ^14^, ^15^, ^16^

This study assesses whether body composition measurements obtained using abdominal CTs are independently associated with 90‐day and 1‐year mortality in patients with long bone metastases undergoing surgery.

## 2 MATERIALS AND METHODS

### 2.1 Study design and setting

This study complied with the Health Insurance Portability and Accountability Act guidelines. Our institutional review board approved a waiver of informed consent for this retrospective study, performed at a tertiary institution between January 1st, 1999 and January 1st, 2017. We adhered to the Strengthening Reporting of Observational Studies in Epidemiology guidelines.^17^

### 2.2 Participants and clinical characteristics

**Figure 1:**
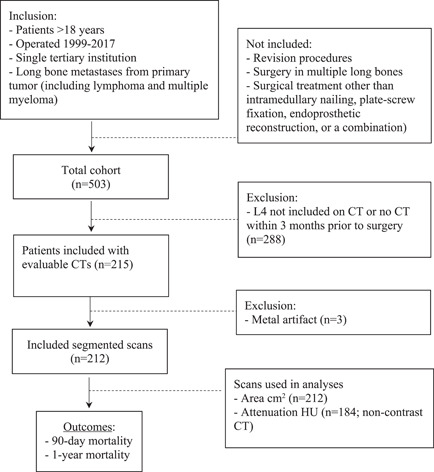
Flow diagram depicting patient selection

This single institutional retrospective study, performed at an urban tertiary care referral center for orthopaedic oncology, included: (1) patients 18 years of age or older, (2) surgery for long bone metastases (inclusive of lymphoma and multiple myeloma), and (3) availability of abdominal CT within 3 months before surgery.^18^ Long bones were defined as femur, humerus, tibia, fibula, radius, and ulna. Excluding criteria were (1) metastatic fractures in multiple bones requiring surgery, (2) revision procedures, (3) surgery other than intramedullary nailing, dynamic hip screw, plate‐screw fixation, endoprosthetic reconstruction, or a combination thereof, (4) L4 not included on abdominal CT, and (5) CT not assessable due to metal artifacts. Choice of treatment was decided by mutual agreement between the patient and surgeon, guided by the Mirels score. Mirels described a scoring system for predicting the likelihood of pathological fracture in 1989. It takes four factors into account: anatomical site, intensity of discomfort, radiographic appearance, and size of metastatic lesion. Each component is assigned a score and the higher the score, the more risk of pathological fracture and hence surgical intervention.^19^ For patients who underwent multiple CTs within 3 months before surgery, only the nearest CT to surgery was included. The first surgery was included if a patient received multiple surgeries meeting the selection criteria. All included CTs were used for determining body composition cross sectional areas (CSA) and only noncontrast CTs for body composition attenuation measurements (Figure 1).^20^

Clinical factors known to be associated with survival^18^ were obtained by manual review of medical charts: age, sex, body mass index (BMI, kg/m^2^), modified Charlson Comorbidity in addition to metastases^21^; primary tumor categorized as slow, moderate or rapid growth as classified by Katagiri et al.,^22^ pathological fracture (actual completed fracture or impending fracture), location of long bone metastases (lower or upper extremity), additional metastases to the metastasis operated for, previous systemic therapy, previous local radiotherapy, type of surgical treatment, duration in days of primary tumor diagnosis until metastatic operation, preoperative albumin level (g/dl) within 2 weeks of the operation, and 30‐day postoperative complications including venous thromboembolism, wound infection and/or dehiscence, myocardial infarction, pneumonia, and sepsis.^23^

### 2.3 CT body composition measurements

The method for body composition assessment were described in detail in our previous study evaluating patients with spinal metastases undergoing surgery.^24^ Briefly, measurements were performed at the level of the 4th lumbar vertebra using an in‐house automated algorithm. The software used by our group was developed in house and is an extension of previously described algorithms.^25^, ^26^, ^27^ This particular methodology for body composition segmentation was also presented at RSNA 2018 (Scientific Session Program Book SSG08‐05). The software is based on Python, Keras/Tensorflow, and utilizes deep convolutional neural networks to provide tissue segmentations that are then scrutinized by a trained member of the research team (CGB) for any necessary corrections, under supervision of two experienced fellowship‐trained, board‐certified musculoskeletal radiologists (MT, MAB). Body composition measurements included CSA and attenuation of three tissues: VAT, SAT, and paraspinal/abdominal muscle. Attenuation measurements represent the radiodensity expressed in HU of tissue and is a qualitative measurement.^12^ Muscle CSA was used to determine sarcopenia using total muscle CSA (cm^2^) divided by the height squared (m^2^), with cutoff values of <52.4 cm^2^/m^2^ for men and <38.5 cm^2^/m^2^ for women based on a meta‐analysis of 7.843 patients from 38 studies.^15^

### 2.4 Outcomes

The outcomes of interest were mortality by any cause after surgery at 90 days and 1 year. Date of death was obtained from medical charts and the Social Security Index.^28^ Loss to follow‐up in survival was 5.2% (11/212) at 90 days and 8.0% (17/212) at 1 year. Follow‐up was verified until May 15th, 2020.

### 2.5 Statistical analysis

Variables are presented as medians with interquartile ranges (IQRs) for continuous variables and frequencies with percentages for categorical variables. Clinical variables are compared between included and excluded patients using the Mann–Whitney *U* test for continuous variables and Chi‐squared test for categorical variables. Nonparametric testing was used for continuous variables as they were not normally distributed based on inspection of histograms.

Bivariate Cox proportional hazard analysis explored associations between clinical and radiologic variables and the 90‐day and 1‐year mortality outcomes. We used multivariate Cox proportional hazard analysis including all variables identified in bivariate testing with a *p* value of <0.10. The Cox proportionality assumptions and collinearity were tested before performing multivariate analyses. Body composition measurements with a *p* value of <0.10 were included separately in the multivariate analyses. Sarcopenia was considered instead of muscle CSA in the multivariate analysis. All body composition measurements were—besides sarcopenia—included as continuous variables in the analyses. The Cox results were presented as hazard ratios (HR) with 95% confidence intervals (CI). Kaplan–Meier plots demonstrated the probability of survival for patients with and without sarcopenia. No sample size was calculated since all eligible patients between 1999 and 2017 were included. Survival did not change during the 18‐year period (94 patients ≤2010 with 66% mortality rate vs. 118 patients ≥2011 with 62% mortality rate; *p* = 0.27) as did sarcopenia (40% vs. 37%; *p* = 0.77). We applied multiple imputations    to estimate missing values for BMI in 8 patients (3.8%), and albumin in 5 patients (2.4%). For all analyses, a two‐sided *p* value of <0.05 was considered significant. All statistical analyses were performed using Stata 15.0 (StataCorp LP).

## 3 RESULTS

### 3.1 Study population

Study patients included 49% (*n* = 103) men and 51% (*n* = 109) women, with a median age of 63 years (IQR: 56–69) and a median\ BMI of 26 kg/m^2^ (IQR: 23–30) (Table 1) who underwent surgery for long bone metastases. Of the 212 surgeries, 76% (*n* = 162) involved the lower extremities and 24% (*n* = 50) the upper extremities. Systemic therapy was administered before surgery in 58% (*n* = 122) and local radiotherapy in 16% (*n* = 33). The four most common primary tumors included lung (22%), renal cell (15%), breast hormone dependent (11%), and multiple myeloma (11%; see Appendix, Supporting Information Digital Content 1). The included patients (*n* = 212) differed from the excluded patients (*n* = 291) in the following four characteristics: more comorbidities, more moderate and rapid primary tumor growth, more additional metastases, and a higher 1‐year mortality rate (see Appendix, Supporting Information Digital Content 2).

**Table 1:** Baseline characteristics of patients treated for long bone metastases (*n *= 212)

Variables	Median (IQR)
Age (years)	63 (56–69)
Body mass index (in kg/m^2^)^a^	26 (23–30)
Duration primary diagnosis until metastatic operation (months)^a^	12 (1–41)
Preoperative albumin (g/dl)	3.7 (3.3–4.1)
	**% (*n*)**
Men	49 (103)
Race	
White	92 (195)
Non‐white	8 (17)
Other Modified Charlson Comorbidity	69 (147)
Primary tumor growth^b^	
Slow	29 (62)
Moderate	29 (61)
Rapid	42 (89)
Additional metastases^c^	87 (185)
Tumor location	
Upper extremity	24 (50)
Lower extremity	76 (162)
Type of surgery	
Intramedullary nail	45 (96)
Endoprosthetic reconstruction	25 (53)
Plate and screw fixation	25 (53)
Dynamic hip screw	2 (4)
Multiple implements	3 (6)
Previous local radiotherapy	16 (33)
Previous systemic therapy	58 (122)
Completed pathological fracture	56 (118)
Mortality^a^	
90 days	32 (64)
1 year	64 (124)
**Body composition measurements**	**Median (IQR)**
Subcutaneous adipose tissue	
Area (cm^2^)	264 (180–351)
Attenuation (HU)	−94 (−101; −87)
Visceral adipose tissue	
Area (cm^2^)	134 (74–186)
Attenuation (HU)	−84 (−90; −69)
Muscle	
Area (cm^2^)	133 (109–158)
Attenuation (HU)	31 (23–36)
	**% (*n*)**
Sarcopenia^d^	39 (79)

Abbreviations: HU, Hounsfield units; IQR, interquartile range; kg/m^2^, kilogram per square meter.

^a^ Body mass index was available in 204 patients (96%), albumin in 207 patients (98%), sarcopenia in 205 patients (97%), 90‐day mortality in 201 patients (95%), and 1‐year mortality in 195 patients (92%).

^b^ Based on histology groupings; slow growth includes hormone dependent breast cancer, hormone dependent prostate cancer malignant lymphoma malignant myeloma, and thyroid cancer; moderate growth includes non‐small cell lung cancer with molecularly targeted therapy, hormone independent breast cancer, hormone independent prostate cancer, renal cell carcinoma, sarcoma, other gynecological cancer, and others; and rapid growth includes other lung cancer, colon and rectal cancer, gastric cancer, hepatocellular carcinoma, pancreatic cancer, head and neck cancer, other urological cancer, esophageal cancer, malignant melanoma, gallbladder cancer, cervical cancer, and unknown origin. Patients with breast and prostate cancer were separated into groups based on their sensitivity to hormone treatment. Patients who had previously received several hormonal therapy agents, or breast cancer patients who lacked both progesterone and estrogen receptors, were deemed hormone independent.

^c^ Any bone metastasis outside of the lesion treated for.

^d^ Sarcopenia cut‐off values were <52.4 cm^2^/m^2^ (males) and <38.5 cm^2^/m^2^ (females).

^e^ These values were based on any additional comorbidity on top of the metastatic disease score according to the modified Charlson Comorbidity Index.

The 90‐days mortality was 32% (*n* = 64) and 1 year 64% (*n* = 124). Median body composition CSA are shown in Table 1. Patients with sarcopenia (*n* = 79) were older and more frequently male compared with patients without sarcopenia (see Appendix, Supporting Information Digital Content 3). No difference was observed in postoperative complication rate between patients with sarcopenia and without sarcopenia (7.6% vs. 15%; *p* = 0.36). The mean survival was 17 months for patients without sarcopenia and 10 months with sarcopenia.

### 3.2 90‐day mortality

Bivariate analysis demonstrated that five clinical variables were associated with increased 90‐day mortality: lower albumin level, non‐white race, comorbidities, rapid primary tumor growth, and previous systemic therapy (all *p* < 0.05). Two body composition measurement were associated with increased 90‐day mortality: presence of sarcopenia and lower muscle attenuation (see Appendix, Supporting Information Digital Content 4). In multivariate analysis after controlling for the five clinical variables, the presence of sarcopenia remained associated with an increased 90‐day mortality (HR: 1.87; 95% CI: 1.11–3.16; *p* = 0.019; Table 2) but not muscle attenuation (HR: 0.98; 95% CI: 0.96–1.00; *p* = 0.079; see Appendix, Supporting Information Digital Content 5).

**Table 2:** Multivariable cox proportional hazard analysis for the risk of 90‐day death after surgery for long bone metastases using pooled imputed data

Variables	Hazard ratio (95% CI)	Standard error	*p* value
Albumin	0.39 (0.26–0.60)	0.083	**<0.001**
Additional Charlson comorbidity	1.61 (0.80–3.22)	0.571	0.183
White	0.46 (0.20–1.06)	0.196	0.068
Primary tumor growth			
Slow	0.22 (0.10–0.49)	0.089	**<0.001**
Moderate	0.54 (0.30–0.99)	0.169	**0.050**
Rapid	*Reference value*		
Previous systemic therapy	1.94 (1.09–3.45)	0.570	**0.024**
Sarcopenia	1.87 (1.11–3.16)	0.499	**0.019**

*Note*: Bold *p* values are <0.05.

Abbreviation: CI, confidence interval.

### 3.3 1‐year mortality

**Figure 2:**
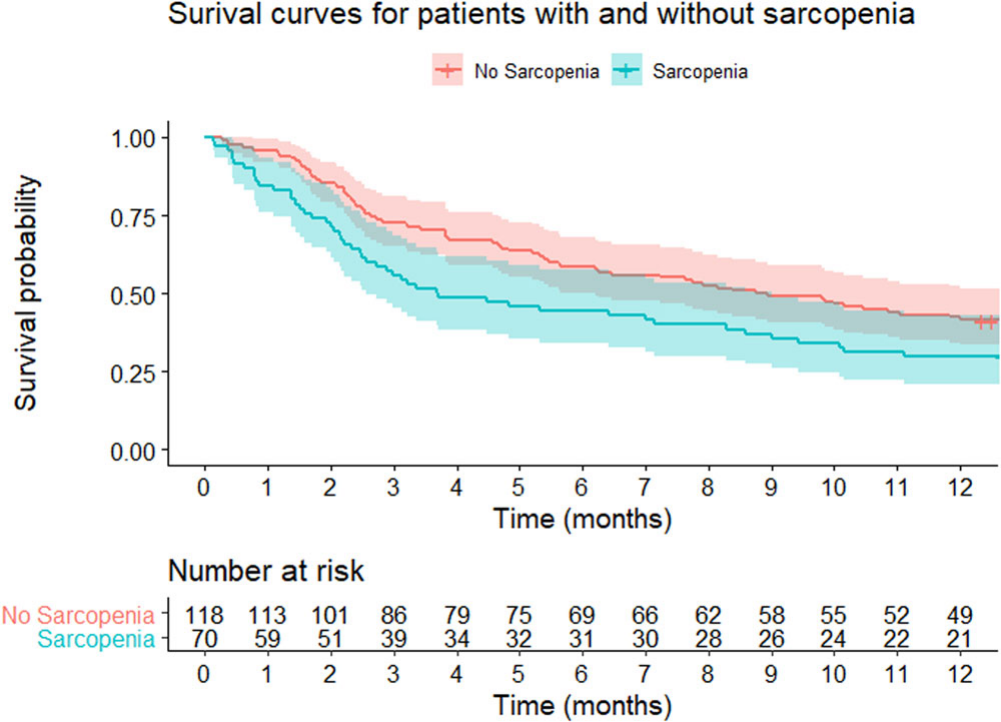
Kaplan–Meier plot showing the survival probability with 95% confidence intervals for patients with and without sarcopenia

Bivariate analysis demonstrated that three clinical variables were associated with increased 1‐year mortality: lower albumin level, presence of comorbidities and rapid primary tumor growth (all *p* < 0.05). In addition, three clinical variables had a *p* value of <0.10: race, additional metastases, and previous systemic therapy. Two body composition measurements were associated with increased 1‐year mortality: muscle CSA and presence of sarcopenia, of which the latter was included in multivariate analysis. In multivariate analysis after controlling for six clinical variables, the presence of sarcopenia remained associated with an increased 1‐year mortality (HR: 1.50; 95% CI: 1.02–2.19; *p* = 0.038; Table 3). The Kaplan–Meier plot illustrated the increased survival probability of patients without sarcopenia (Figure 2).

**Table 3:** Multivariable cox proportional hazard analysis for the risk of 1‐year death after surgery for long bone metastases using pooled imputed data

Variables	Hazard ratio (95% CI)	Standard‐error	*p* value
Albumin	0.41 (0.30–0.55)	0.063	**<0.001**
Additional Charlson comorbidity	1.54 (0.98–2.42)	0.354	0.060
White	0.41 (0.20–0.83)	0.149	**0.014**
Primary tumor growth			
Slow	0.20 (0.12–0.33)	0.053	**<0.001**
Moderate	0.38 (0.24–0.60)	0.088	**<0.001**
Rapid	*Reference value*		
Previous systemic therapy	1.35 (0.89–2.04)	0.287	0.163
Additional metastases	1.85 (0.89–3.85)	0.692	0.102
Sarcopenia	1.50 (1.02–2.19)	0.292	**0.038**

*Note*: Bold *p* values are <0.05.

Abbreviation: CI, confidence interval.

## 4 DISCUSSION

Survival prognostication is an important element in the surgical decision‐making process for patients with long‐bone metastases.^9^, ^10^, ^18^ Various survival prediction tools have been developed for patients with cancer,^1^, ^7^, ^9^, ^18^, ^29^ but these tools are limited as all clinical factors might not be available.^10^ Studies concerning patients with extremity sarcoma,^13^ spinal metastases,^12^, ^30^, ^31^ and nonosseous malignant neoplasms^32^, ^33^, ^34^, ^35^ have identified body composition measurements derived from CTs performed for other purposes (opportunistic CT) as predictor of survival. Our study demonstrates that the presence of sarcopenia is associated with both 90‐day and 1‐year mortality while muscle attenuation, SAT and VAT (both attenuation and CSA) were not associated with mortality. To our knowledge, this is the first study assessing CT body composition measurements as predictors of survival in patients surgically treated for long bone metastases while controlling for multiple clinical variables.

Sarcopenia, or the involuntary loss of skeletal muscle, has been associated with risk for mortality in patients with various primary malignant neoplasms such as pancreatic, gastric, breast, and lung cancer, in addition to patients suffering from metastatic disease.^36^, ^37^, ^38^ The underlying mechanism that links sarcopenia to mortality in patients with malignant disease has not been well defined. Various candidate mechanisms for muscle wasting have been described—ranging from muscle catabolism due to systemic inflammation, to the inhibition of myoblast differentiation caused by an uninhibited release of the negative muscle cell differentiation regulator, myostatin.^39^ However, the involuntary muscle loss is most likely attributed to several simultaneously acting molecular pathways.

The outcomes of this study may have potential implications for clinical care and research. First, our results of the association with sarcopenia with mortality could help clinicians and patients in the shared decision‐making process. By integrating the automatically collected body composition biomarker into the electronic health record, clinicians gain yet another aid to better determine optimal treatment for the patient. Future prediction tools can implement both body composition measures and clinical factors to identify patients with poor survival outcome. We do not advocate that patients with sarcopenia should be withheld surgery. Instead, patients together with clinicians can be guided with robust prognostic information on what to expect if they opt for surgical treatment. To benefit clinical oncologic practice, future research should also include nonoperative patients to improve treatment selection of patients with bone metastases. With this, patients who may benefit from surgical treatment or nonsurgical treatment can be identified. Second, the finding that the presence of sarcopenia is related to poor survival for both time‐points, suggests that involuntary weight loss is not a surrogate for skeletal muscle depletion. Brown et al.^40^ showed that, despite body weight stability over time, 1 in 8 patients with colorectal cancer developed incident sarcopenia. Other studies have previously suggested that frailty is better indicated by skeletal muscle loss than decreased body weight.^30^, ^41^ In other words, the absence of a history of weight loss does not rule out skeletal muscle depletion and the use of CTs for body composition analysis should be considered more broadly as a part of clinical care. Third, apart from the outcomes concerning sarcopenia, primary tumor growth and decreased albumin were found to be independent predictors of a higher risk of mortality in this study. Because these two additional predictors have been successfully incorporated in prognostication tools in previous studies,^7^ adding the presence of sarcopenia as a variable to these tools may strengthen these predictions.

This study must be interpreted in light of its limitations. First, only patients who had undergone a CT scan which included the L4 vertebrae were included for analysis. This resulted in the exclusion of 58% of patients (291 of 503) that were surgically treated for long bone metastases, which may be a source of potential bias. Upon comparison of the patients with and without available CT, we found that patients in the non‐CT group had fewer additional Charlson comorbidities, less additional metastases, more primary tumor types with slow growth, and lower 1‐year mortality. This suggests that patients without available CT generally consisted of healthier patients with less advanced disease. The prevalence of sarcopenia in this group is unknown which may have impacted the results of this study. However, we believe that this issue has minor impact on the results of this study as we have seen a clear link between sarcopenia and mortality in the frailer patient population included for analysis. Another limitation was that only 42% patients had CTs available that met inclusion criteria. Of note, the patient without CTs were predominately from the earlier years (before 2010). With more access to imaging and greater emphasis of using the CT for staging and surveillance of cancer as well as preoperative work up, we predict that most patients will have CTs available that can be used for body composition assessment. Second, there were several factors for which we could not control for in multivariate analysis, such as the Eastern Cooperative Oncology Group performance status,^42^ and preoperative quality of life measures. These measures indicate the preoperative ambulatory status, which may be linked to the amount of skeletal muscle in the patient. Future studies should include these measures to reevaluate and validate these findings. Third, we did not perform analyses of other important secondary outcomes for patients with long bone metastases such as quality of life changes, reoperations, and length of hospitalization. Evidence exists in literature concerning nonosseous neoplasms that body composition measurements has predictive value in these secondary outcomes.^32^, ^43^ Fourth, even though metastases from malignant lymphoma (5 patients with no sarcopenia and 4 patients with sarcopenia) and multiple myeloma (14 patients with no sarcopenia and 9 patients with sarcopenia) are known for their better prognosis, we did include these cases as they formed 16% (33 of 212) of the study cohort. Fifth, the measurements are done by an “in house” mechanism which limits generalized use of our methods. However, the measurements performed by the software are generalizable, as they are standard body composition measures, which are typically performed manually or semiautomated. The motivation of using our automated software was to reduce the time burden for performing these measurements. Of note, all measurements were checked by a trained research analysts and radiologists to confirm that measurements were performed correctly. Last, this study encompasses 18 years during which time (systemic) management has changed for extremity metastatic disease. However, the survival did not change during this period as did sarcopenia.

## 5 CONCLUSIONS

The presence of sarcopenia assessed by CT is predictive of 90‐day and 1‐year mortality for patients undergoing surgery for long bone metastases, independent of established risk factors. The presence of sarcopenia could serve as novel biomarker to be included in prediction tools. Future studies should investigate the added benefit of sarcopenia and other opportunistic CT body composition measures to existing prognostic tools. Accurate and reliable survival prediction is crucial to improve shared decision making for patients with long bone metastases that are considering surgical management.

## CONFLICT OF INTERESTS

The authors declare that there are no conflict of interests.

## ETHICS STATEMENT

This study was approved by our institutional review board

## SYNOPSIS

This single institutional retrospective study included 212 patients who had undergone surgery for long‐bone metastases and had a CT of the abdomen within 90 days before surgery. The presence of sarcopenia assessed by CT was predictive of 90‐day and 1‐year mortality.

## Supporting information

Supporting information.

Supporting information.

Supporting information.

Supporting information.

Supporting information.

## Data Availability

Data available on request due to privacy/ethical restrictions. Miriam A. Bredella Division of Musculoskeletal Imaging and Intervention Department of Radiology, Massachusetts General Hospital and Harvard Medical School Yawkey 6E, 55 Fruit Street, Boston, MA 02114, USA.
