# Supplementary material for: Body composition predictors of mortality in patients undergoing surgery for long bone metastases
Source: J Surg Oncol. 2022 Jan 13;125(5):916–23. doi: 10.1002/jso.26793 (PMC8917991; doi:10.1002/jso.26793)
Supplement: Supplementary file 1 — Supporting information. [file JSO-125-916-s004.docx]

| **Supplementary table 1**. Origin of primary tumor (n=212) | |
| --- | --- |
| ***Primary tumor*** | ***% (n)*** |
| Lung | 22 (47) |
| Renal cell | 15 (32) |
| Breast hormone dependent | 11 (24) |
| Multiple myeloma | 11 (23) |
| Non-small-cell lung targeted therapy | 4.7 (10) |
| Esophageal | 4.7 (10) |
| Malignant lymphoma | 4.7 (10) |
| Breast hormone independent | 3.7 (8) |
| Melanoma | 3.7 (8) |
| Hepatocellular | 3.3 (7) |
| Head and neck | 2.8 (6) |
| Unknown origin | 2.4 (5) |
| Other | 1.9 (4) |
| Colon and rectal | 1.4 (3) |
| Prostate hormone independent | 1.4 (3) |
| Thyroid | 1.4 (3) |
| Prostate hormone dependent | 0.9 (2) |
| Pancreatic | 0.9 (2) |
| Sarcoma | 0.9 (2) |
| Other urological | 0.9 (2) |
| Gastric | 0.5 (1) |
