# Supplementary material for: Body composition predictors of mortality in patients undergoing surgery for long bone metastases
Source: J Surg Oncol. 2022 Jan 13;125(5):916–23. doi: 10.1002/jso.26793 (PMC8917991; doi:10.1002/jso.26793)
Supplement: Supplementary file 2 — Supporting information. [file JSO-125-916-s003.docx]

| **Supplementary table 2.** Comparison between included (n=212) and excluded, non-CT group (n=291) of patients surgically treated for long bone metastases (n=503). | | | |
| --- | --- | --- | --- |
| ***Variables*** | ***Included (n=212)*** | ***Excluded (n=291)*** | ***p-value*** |
|  | ***Median (IQR)*** | ***Median (IQR)*** |  |
| Age (years) | 63 (56-69) | 65 (54-74) | 0.13 |
| Body mass index (in kg/m^2^)^a^ | 26 (23-30) | 27 (24-31) | 0.21 |
| Duration primary diagnosis till metastatic operation (months) | 12 (1-41) | 15 (1-65) | 0.15 |
| Albumin (g/dL)^a^ | 3.7 (3.3-4.1) | 3.6 (3.2-4.1) | 0.33 |
|  | ***% (n)*** | ***% (n)*** |  |
| Male | 49 (103) | 45 (131) | 0.16 |
| Race^a^ |  |  | 0.87 |
| White | 92 (117) | 92 (263) |  |
| Non-white | 8.0 (17) | 7.7 (22) |  |
| Additional Modified Charlson Comorbidity | 69 (147) | 47 (137) | **<0.01** |
| Primary tumor growth |  |  | **<0.01** |
| Slow | 29 (62) | 49 (143) |  |
| Moderate | 29 (61) | 20 (57) |  |
| Rapid | 42 (89) | 31 (91) |  |
| Additional metastases | 87 (185) | 77 (225) | **0.01** |
| Location of bone metastases |  |  | 0.88 |
| Upper extremity | 24 (50) | 23 (67) |  |
| Lower extremity | 76 (162) | 77 (224) |  |
| Type of surgery |  |  | 0.33 |
| Intramedullary nail | 45 (96) | 40 (117) |  |
| Endoprosthetic reconstruction | 25 (53) | 29 (83) |  |
| Plate and screw fixation | 25 (53) | 26 (75) |  |
| Dynamic hip screw | 2 (4) | 4.1 (12) |  |
| Multiple implements | 3 (6) | 1.4 (4) |  |
| Previous local radiotherapy | 16 (33) | 18 (52) | 0.50 |
| Previous systemic therapy | 58 (122) | 53 (153) | 0.27 |
| Completed pathological fracture | 57 (118) | 59 (172) | 0.44 |
| Mortality^a^ |  |  |  |
| 90-days | 32 (64) | 24 (66) | 0.08 |
| 1 year | 64 (124) | 54 (144) | **0.05** |
| IQR=Interquartile range; kg/m^2^=kilogram per square meter. P-values are calculated with the t-test for continuous variables, the Chi-squared test for categorical variables, and Cox proportional hazard model for survival. **Bold** p-values are <0.05. a Missing values of the included group are listed in Table 1. For the excluded group, body mass index was available in 198 patients (68%), albumin in 210 patients (72%), race in 285 patients (98%), 90-day mortality in 276 patients (95%), and 1-year mortality in 267 patients (92%). | | | |
