# Supplementary material for: Body composition predictors of mortality in patients undergoing surgery for long bone metastases
Source: J Surg Oncol. 2022 Jan 13;125(5):916–23. doi: 10.1002/jso.26793 (PMC8917991; doi:10.1002/jso.26793)
Supplement: Supplementary file 3 — Supporting information. [file JSO-125-916-s002.docx]

| **Supplementary table 3.** Comparison between sarcopenia and no sarcopenia. | | |  |
| --- | --- | --- | --- |
| **Variables** | **No Sarcopenia** | **Sarcopenia** | **p-value** |
|  | ***Median (IQR)*** | ***Median (IQR)*** |  |
| Age (years) | 61 (53-66) | 66 (60-74) | **<0.01** |
| Body mass index (in kg/m^2^) | 28 (25-32) | 24 (21-27) | **<0.01** |
| Duration primary diagnosis till metastatic operation (months) | 12 (1-40) | 13 (1-42) | 0.98 |
| Preoperative albumin (g/dL) | 3.8 (3.3-4.1) | 3.8 (3.2-4.2) | 0.83 |
|  | *% (n)* | *% (n)* |  |
| Male | 37 (47) | 71 (56) | **<0.01** |
| Race |  |  | 0.30 |
| White | 94 (118) | 89 (70) |  |
| Non-white | 6.4 (8) | 11 (9) |  |
| Additional Modified Charlson Comorbidity | 69 (87) | 68 (54) | 0.99 |
| Primary tumor growth |  |  | 0.65 |
| Slow | 32 (40) | 27 (21) |  |
| Moderate | 29 (36) | 28 (22) |  |
| Rapid | 40 (50) | 46 (36) |  |
| Additional metastases | 86 (108) | 91 (72) | 0.28 |
| Metastases location |  |  | 0.74 |
| Upper extremity | 23 (29) | 25 (20) |  |
| Lower extremity | 77 (97) | 75 (59) |  |
| Type of surgery |  |  | 0.98 |
| Intramedullary nail | 44 (56) | 47 (37) |  |
| Endoprosthetic reconstruction | 25 (32) | 23 (18) |  |
| Plate and screw fixation | 25 (31) | 27 (21) |  |
| Dynamic hip screw | 2.4 (3) | 1.3 (1) |  |
| Multiple implements | 3.2 (4) | 2.5 (2) |  |
| Previous local radiotherapy | 17 (22) | 14 (11) | 0.56 |
| Previous systemic therapy | 63 (79) | 53 (42) | 0.19 |
| Pathologic fracture | 49 (62) | 63 (50) | 0.06 |
| Complications within 30 days | 12 (15) | 7.6 (6) | 0.36 |
| Mortality |  |  |  |
| 90 days | 26 (32) | 42 (30) | **0.03** |
| 1 year | 58 (69) | 70 (49) | **0.04** |
| IQR = Interquartile range; kg/m^2^ = kilogram per square meter. P-values are calculated with the (1) t-test for continuous, (2) the Chi-squared test for categorical variables, and (3) Cox proportional hazard model for survival. Bold p-values are <0.05. Missing values are listed in Table 1. | | | |
