# Supplementary material for: Body composition predictors of mortality in patients undergoing surgery for long bone metastases
Source: J Surg Oncol. 2022 Jan 13;125(5):916–23. doi: 10.1002/jso.26793 (PMC8917991; doi:10.1002/jso.26793)
Supplement: Supplementary file 4 — Supporting information. [file JSO-125-916-s001.docx]

| **Supplementary table 4.** Bivariate cox proportional hazard analysis for the risk of death (90-days and 1-year) in long bone metastases (n=212) after multiple imputation (n=40). | | | | | | |
| --- | --- | --- | --- | --- | --- | --- |
|  | **90 days** | | | **1-year** | | |
| ***Variables*** | ***Hazard ratio (95% CI)*** | ***SE*** | ***p-value*** | ***Hazard ratio (95% CI)*** | ***SE*** | ***p-value*** |
| Age (years) | 1.02 (0.99-1.04) | 0.011 | 0.179 | 1.01 (0.99-1.02) | 0.008 | 0.421 |
| Body mass index (in kg/m^2^) | 0.99 (0.94-1.03) | 0.023 | 0.529 | 0.98 (0.95-1.01) | 0.016 | 0.206 |
| Duration primary diagnosis until metastatic operation (months) | 1.00 (1.00-1.00) | 0.001 | 0.569 | 0.99 (0.99-1.01) | 0.001 | 0.226 |
| Albumin | 0.41 (0.29-0.59) | 0.076 | **<0.001** | 0.49 (0.38-0.64) | 0.066 | **<0.001** |
| Male | 1.01 (0.61-1.63) | 0.250 | 0.999 | 1.09 (0.77-1.55) | 0.196 | 0.623 |
| White | 0.45 (0.21-0.99) | 0.182 | **0.049** | 0.54 (0.27-1.06) | 0.186 | 0.073 |
| Other Modified Charlson Comorbidity | 2.24 (1.17-4.30) | 0.744 | **0.015** | 2.04 (1.33-3.14) | 0.447 | **0.001** |
| Primary tumor growth |  |  |  |  |  |  |
| Slow | 0.28 (0.13-0.57) | 0.102 | **0.001** | 0.28 (0.17-0.44) | 0.067 | **<0.001** |
| Moderate | 0.55 (0.31-0.98) | 0.163 | **0.044** | 0.46 (0.30-0.70) | 0.099 | **<0.001** |
| Rapid | *Reference value* | | | *Reference value* | | |
| Additional metastases* | 1.90 (0.76-4.74) | 0.885 | 0.168 | 1.85 (0.99-3.43) | 0.583 | 0.053 |
| Tumor location |  |  |  |  |  |  |
| Upper extremity | 1.38 (0.80-2.39) | 0.385 | 0.242 | 1.05 (0.69-1.60) | 0.226 | 0.816 |
| Lower extremity | *Reference value* | | | *Reference value* | | |
| Type of surgery |  |  |  |  |  |  |
| Intramedullary nail | *Reference value* | | | *Reference value* | | |
| Endoprosthetic | 0.84 (0.44-1.61) | 0.280 | 0.599 | 0.85 (0.55-1.33) | 0.194 | 0.487 |
| Plate and screw fixation | 1.06 (0.58-1.93) | 0.324 | 0.851 | 0.98 (0.63-1.51) | 0.217 | 0.915 |
| Dynamic hip screw | 2.17 (0.52-9.11) | 1.589 | 0.289 | 0.91 (0.22-3.73) | 0.655 | 0.896 |
| Multiple implants | 1.88 (0.57-6.17) | 1.141 | 0.298 | 1.30 (0.47-3.59) | 0.673 | 0.610 |
| Previous local radiotherapy | 1.20 (0.64-2.25) | 0.384 | 0.571 | 1.12 (0.70-1.79) | 0.269 | 0.632 |
| Previous systemic therapy | 1.75 (1.03-2.97) | 0.472 | **0.038** | 1.39 (0.97-2.01) | 0.261 | 0.076 |
| Completed pathological fracture | 1.40 (0.85-2.32) | 0.359 | 0.187 | 1.28 (0.90-1.83) | 0.233 | 0.175 |
| ***Body composition measurements*** |  |  |  |  |  |  |
| Subcutaneous adipose tissue |  |  |  |  |  |  |
| Area (cm^2^) | 0.99 (0.99-1.01) | 0.001 | 0.484 | 0.99 (0.99-1.01) | 0.001 | 0.329 |
| Attenuation (HU) | 1.01 (0.99-1.02) | 0.008 | 0.234 | 0.99 (0.99-1.01) | 0.006 | 0.709 |
| Visceral adipose tissue |  |  |  |  |  |  |
| Area (cm^2^) | 0.99 (0.99-1.01) | 0.001 | 0.713 | 1.00 (0.99-1.01) | 0.001 | 0.261 |
| Attenuation (HU) | 0.99 (0.99-1.02) | 0.008 | 0.862 | 1.00 (099-1.01) | 0.006 | 0.508 |
| Muscle |  |  |  |  |  |  |
| Area (cm^2^) | 0.99 (0.98-1.01) | 0.004 | 0.067 | 0.99 (0.98-0.99) | 0.003 | **0.043** |
| Attenuation (HU) | 0.98 (0.96-1.01) | 0.012 | 0.077 | 0.99 (0.97-1.01) | 0.009 | 0.158 |
| Sarcopenia | 1.76 (1.07-2.90) | 0.449 | **0.026** | 1.46 (1.01-2.11) | 0.274 | **0.042** |
| *IQR=Interquartile range; kg/m^2^=kilogram per square meter; CI=confidence interval; SE=Standard error; HU=Hounsfield units.* ***Bold*** *p-values are <0.05.*  **No differences were found in additional analyses with the variables brain metastases (yes/no), visceral metastases (yes/no) and bone metastases (yes/no).* | | | | | | |
