# Supplementary material for: Body composition predictors of mortality in patients undergoing surgery for long bone metastases
Source: J Surg Oncol. 2022 Jan 13;125(5):916–23. doi: 10.1002/jso.26793 (PMC8917991; doi:10.1002/jso.26793)
Supplement: Supplementary file 5 — Supporting information. [file JSO-125-916-s005.docx]

| **Supplementary table 5**. Multivariable cox proportional hazard analysis for the risk of 90-day mortality for muscle attenuation after surgery for long bone metastases using pooled imputed data. | | | |
| --- | --- | --- | --- |
| ***Variables*** | ***Hazard ratio (95% CI)*** | ***Standard-error*** | ***p-value*** |
| Albumin | 0.40 (0.26-0.63) | 0.093 | **<0.001** |
| Additional Charlson comorbidity | 1.48 (0.73-3.01) | 0.536 | 0.278 |
| White | 0.35 (0.15-0.80) | 0.147 | **0.012** |
| Primary tumor growth |  |  |  |
| Slow | 0.26 (0.11-0.59) | 0.108 | **0.001** |
| Moderate | 0.60 (0.31-1.15) | 0.200 | 0.125 |
| Rapid | *Reference value* | | |
| Previous systemic therapy | 2.21 (1.20-4.06) | 0.686 | **0.011** |
| Muscle attenuation | 0.98 (0.96-1.00) | 0.012 | 0.079 |
| CI=confidence interval. **Bold** p-values are <0.05. | | | |
